# Combined Enzymatic and Physical Deinking Methodology for Efficient Eco-Friendly Recycling of Old Newsprint

**DOI:** 10.1371/journal.pone.0072346

**Published:** 2013-08-15

**Authors:** Antar Puneet Virk, Minakshi Puri, Vijaya Gupta, Neena Capalash, Prince Sharma

**Affiliations:** 1 Department of Biotechnology, Panjab University, Chandigarh, India; 2 Department of Microbiology, Panjab University, Chandigarh, India; Oak Ridge National Laboratory, United States of America

## Abstract

**Background:**

The development in the deinking process has made recycled fiber a major part of the raw material for pulp and paper industry. Enzymes have revolutionized the deinking process obtaining brightness levels surpassing conventional deinking processes. This study explores the deinking efficiencies of bacterial alkalophilic laccase (L) and xylanase (X) enzymes along with physical deinking methods of microwaving (MW) and sonication (S) for recycling of old newsprint (ONP).

**Methods and Results:**

The operational parameters viz. enzyme dose, pH and treatment time for X and L deinking were optimized statistically using Response Surface Methodology. Laccase did not require any mediator supplementation for deinking. Deinking of ONP pulp with a combination of xylanase and laccase enzymes was investigated, and fiber surface composition and morphological changes were studied using X-ray diffraction, fourier transform infrared spectroscopy and scanning electron microscopy. Compared to the pulp deinked with xylanase (47.9%) or laccase (62.2%) individually, the percentage reduction of effective residual ink concentration (ERIC) was higher for the combined xylanase/laccase-deinked pulp (65.8%). An increase in brightness (21.6%), breaking length (16.5%), burst factor (4.2%) tear factor (6.9%), viscosity (13%) and cellulose crystallinity (10.3%) along with decrease in kappa number (22%) and chemical consumption (50%) were also observed. Surface appeared more fibrillar along with changes in surface functional groups. A combination of physical and enzymatic processes (S-MW-XL) for deinking further improved brightness (28.8%) and decreased ERIC (73.9%) substantially.

**Conclusion:**

This is the first report on deinking of ONP with laccase without any mediator supplementation. XL pretreatment resulted in marked improvement in paper quality and a new sequence being reported for deinking (S-MW-XL) will contribute further in decreasing chemical consumption and making the process commercially feasible.

## Introduction

Recycling of wastepaper has gained momentum over the past decades due to the severity in the demand of green plants being imposed by the paper industry throughout the world [1]. Deinking is an important step in the recycling process and involves the dislodgement of ink particles from fiber surface and then removal of the detached ink particles by flotation, washing etc [2]. The developments in the deinking process have immensely helped the utilization of secondary fiber such as old newsprint, xeroxed papers and laser/inkjet printed papers for making white grade papers. Out of the two types of inks present in secondary fibers, it is easier to remove impact ink such as the one in old newsprint as compared to the non-impact ink present in xeroxed or laser/inkjet printed papers.

The conventional methods of deinking utilize chemicals such as sodium hydroxide, sodium silicate, hydrogen peroxide, chlorine based chemicals and other chelating agents which are environmentally hazardous [3]. Enzymatic deinking has attracted enormous attention by reducing the consumption of chemicals thereby lowering the process cost and making it eco-friendly [2]. Enzymes can act either directly on the fiber or on the ink. Different enzymes being employed for deinking include cellulases, xylanases, pectinases, amylases, lipases, esterases and laccases [4]. The enzymatic deinking using cellulases and hemicellulases alone and in combination has been well characterized [5]. Owing to their action on the cellulosic content of the paper lowering the strength properties, it is desirable to search for more effective enzymes. Laccases are oxidative enzymes that influence the pulp and paper industry in several ways [6]. The role of laccases has been well established in the delignification of pulps resulting in an increase in viscosity [7], [8]. Hence, laccases could be proposed as interesting alternative for deinking of old newsprint which chiefly contains lignin rich mechanical pulp. The detachment of ink particles as well as contaminants, with additional chemical modifications induced by laccase enzyme, could enhance brightness and reduce residual ink concentration [9]. There are reports on use of laccase-mediator systems alone and in combination with cellulases and hemicellulases [10], [2]. The laccases used till date for deinking purposes have been obtained from either fungal sources [5] or plants [9].

Physical methods such as sonication and high temperature are pretreatment stages for deinking owing to their ability to reduce chemical consumption and enhance physical as well as optical properties of recycled papers [11]. As compared to the conventional physical methods using conduction/convection heating, microwaving allows direct interaction between a heated object and an applied electromagnetic field to create volumetric and rapid heating which causes an explosion effect among particles, and improves disruption of recalcitrant structures [12]. Microwave pretreatment was used previously for the biobleaching of kraft pulp in our laboratory [13], and was used for deinking purposes in this work.

This study employed bacterial laccase and xylanase enzymes, sonication and microwaving, alone and in combination, for the deinking of ONP pulp. The parameters for enzymatic deinking were optimized statistically. The optical and physico-mechanical properties of deinked pulp and reduction in chemical consumption were evaluated. The surface properties of enzymatically treated pulp were studied by X-ray diffraction, fourier transform infrared spectroscopy and scanning electron microscopy.

## Materials and Methods

### Wastepaper Pulps

The different pulps used were made from locally procured wastepapers viz. old newsprint, magazines, laser, inkjet and xerox with initial brightness of 36, 42, 65, 60 and 58% ISO respectively.

### Enzymes

A cellulase-free, thermo-alkali-stable and halo-tolerant xylanase with optimum activity at pH 8–9.5 and 65°C was produced from *Bacillus halodurans* FNP 135 soil isolate by solid state fermentation of wheat bran under optimized conditions viz. 2.31% Na_2_CO_3_ (w/w), 80% moisture content (v/w), 68 h of fermentation at 37°C and assayed using birchwood xylan as substrate [14]. One unit activity was defined as the amount of enzyme that produced 1 µmol of reducing sugar (expressed as xylose) per min.

A cellulase and xylanase-free laccase with optimum activity at pH 8 and 55°C was produced from *gamma-proteobacterium,* a novel bacterium isolated from industrial effluent [15] and later identified as a new *Rheinheimera* species [16]. One ml of overnight culture was used to inoculate 100 ml of M162 medium [17] containing 100 µM copper and incubated at 37°C at 150 rpm for 48 h. The culture was centrifuged at 10,000×*g*, 4°C for 10 min and the supernatant was used as crude enzyme preparation. Laccase activity was determined using 2 mM guaiacol, at 55°C in 0.1 M phosphate buffer (pH 6.5) [15]. The change in absorbance due to oxidation of substrate in the reaction mixture was monitored after 10 min of incubation at 465 nm (ε = 48000 M^−1^cm^−1^). Unit of laccase was expressed in nkat (n moles of substrate converted/sec/ml of enzyme).

### Pulp Preparation

Waste papers were shredded and soaked overnight in tap water at room temperature. The soaked papers were washed several times and were disintegrated with the help of a grinder to obtain soft cottony pulp. It was then squeezed to remove absorbed water and oven dried. This oven dried pulp (odp) was used for further experiments.

### Pulp Deinking Sequences

All treatments were carried out in triplicates at least. A standard deinking sequence (P-C) was performed in the laboratory. P refers to the pulp preparation stage and C refers to chemical bleaching stage where pulp at 5% consistency was treated with sodium hydroxide (2%), sodium silicate (2.5%), DTPA (Diethylene triamine penta-acetate, 0.5%), Triton X-100 (1.2%) and H_2_O_2_ (1%) at 50°C and 200 rpm for 15 min. To facilitate removal of ink particles detached from fibers, pulp was thoroughly washed with distilled water and was used to make hand sheets to determine brightness of the pulp.

Enzymatic and physical pretreatments were evaluated using the standard sequence (P-C) by incorporating (a) xylanase (X) stage (P-X-C), (b) laccase (L) stage (P-L-C), (c) L stage along with mediators/inhibitors (M/I) (P-L+M/I-C), (d) X followed by L stage (P-X-L-C), (e) sonication followed by XL stage (P-S-X-L-C), (f) microwaving (MW) followed by XL stage (P-MW-X-L-C), and (g) S followed by MW followed by XL stage (P-S-MW-X-L-C). In all cases, standard sequence (P-C) was used as a control. Both xylanase and laccase treatments were carried out with 10 g (dry weight) of pulp at 5% consistency at different enzyme doses, temperatures and pH for optimization of various parameters for deinking using Response Surface Methodology. The treatments were carried out in flasks, at 160 rev/min and 65°C and 55°C for xylanase and laccase respectively. Laccase mediator and inhibitor treatments were carried out with 10 g (dry weight) of pulp at 5% consistency at optimum pH, enzyme dose and incubation time using 2 mM of 2,2′-azino-bis-3-ethylbenzothiazoline-6-sulphonic acid (ABTS), 1-hydroxybenzotriazole (HBT), syringaldehyde (Syr) as mediators and 1 mM of sodium azide (SA) as inhibitor. Sonication [11] was carried out at 1% consistency, 20 kHz and microwaving [13] was carried out at 20% consistency, 850 W employing optimum pH, enzyme dose and incubation time for different time intervals.

### Analysis of Collected Filtrate

The color removal from the pulp was determined spectrophotometrically from λ 200 nm to λ 800 nm. The reducing sugar released was measured by the dinitrosalicylic acid (DNS) [18], phenolic compounds generated were estimated by measuring the absorbance at λ 237 nm and release of hydrophobic compounds were determined by measuring the absorbance at λ 465 nm [19], [20].

### X-ray Diffraction

Crystallinity of cellulose was determined for both untreated and XL treated samples by X-ray diffraction. After freeze drying, samples were analyzed using the X-ray diffractometer with X-ray generator from 2 to 50 of 2θ (scattering angle). The crystalline index of cellulose (X_c_) was calculated from the X-ray diffraction patterns by the following equation [21].
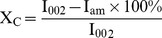



Where I_0 0 2_ is the peak intensity from the (0 0 2) lattice plane (2θ = 22.6°) and I_am_ is the peak intensity of amorphous phase (2θ = 19.0°). Apparent crystallite size (ACS) was estimated through the use of the Scherrer equation [21].
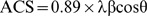
where λ is the wavelength of the incident X-ray (1.5418 Å), θ the Bragg angle corresponding to the (002) plane, and β the half-height width of the peak angle of the (002) reflection.

### Fourier Transformed Infrared Spectroscopy (FTIR)

FTIR spectra for pulp samples were recorded with a resolution of 4 cm^−1^ over the wave number range of 4000–400 cm^−1^, using 32 scans per sample. Empirical cellulose crystallinity index (CI) was calculated by dividing absorbance intensities of IR bands at 1430 and 897 cm^−1^ [22].
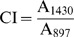



FTIR spectrum was analyzed using the FTIR database [23].

### Scanning Electron Microscopy of Pulp Fibers

The fibers were washed thrice with deionized water and were gradually dehydrated with acetone gradient between 30 and 90% and finally suspended in 100% acetone; small pieces of fibers were air dried and placed on the stubs, mounted with silver tape, and sputter coated with gold using fine coat (JEOL ion sputter, Model JFC-1100) and examined at 10 KV.

### Physical and Chemical Characterization of Hand Sheets

All physical, chemical and residual chlorine measurements were carried out by following the TAPPI standard methods for brightness and whiteness (T-452 OM-87 and T-1216 OM respectively with a Minolta spectrophotometer CM3630), kappa number (T 236), tear factor (T-414 OM-98), burst factor (T-403 OM-97), breaking length (T-404 OM-92) and viscosity (T-230 OM-82). The effective residual ink concentration was measured with the Elrepho ERIC tester using Lorentzen and Wettre software.

## Results and Discussion

### Optimization of Deinking of ONP Pulp with Xylanase (P-X-C) and Laccase (P-L-C) Using Response Surface Methodology

Central composite design with three factors - pH, enzyme dose and incubation time at five levels was employed to investigate the first and higher order main effects of each factor and interaction amongst them for deinking of ONP pulp using xylanase and laccase enzymes (Table S1). With xylanase, maximum brightness of 53.5±0.4% ISO (9% increase) at temperature 65°C, pH 9.0, enzyme dose 15 U/g odp, 150 rpm and incubation time 3 h was obtained; With laccase, maximum brightness of 54.9±0.5% ISO (12% increase) at temperature 55°C, pH 8.0, enzyme dose 50 U/g odp, 150 rpm and incubation time 4 h was obtained (Table S2). This is in accordance with previous studies which showed a brightness increase of 3.22 and 1.7% [10] and 19.4 and 7.8% [5] after xylanase and laccase-mediator pretreatments respectively. ANOVA results of the data disclosed that the models, all independent variables, interactions among pH, enzyme dose as well as time were significant and the lack of fit was not significant (Table S3). The 3D graphs showed that brightness of deinked pulp was affected by the variation of two factors at a time keeping the third at optimal condition in both xylanase and laccase pretreatments and maximum brightness was obtained when all three factors were at their optimal levels (Figure S1 and S2).

### Mediator Supplementation for Deinking with Laccase

**Figure 1:**
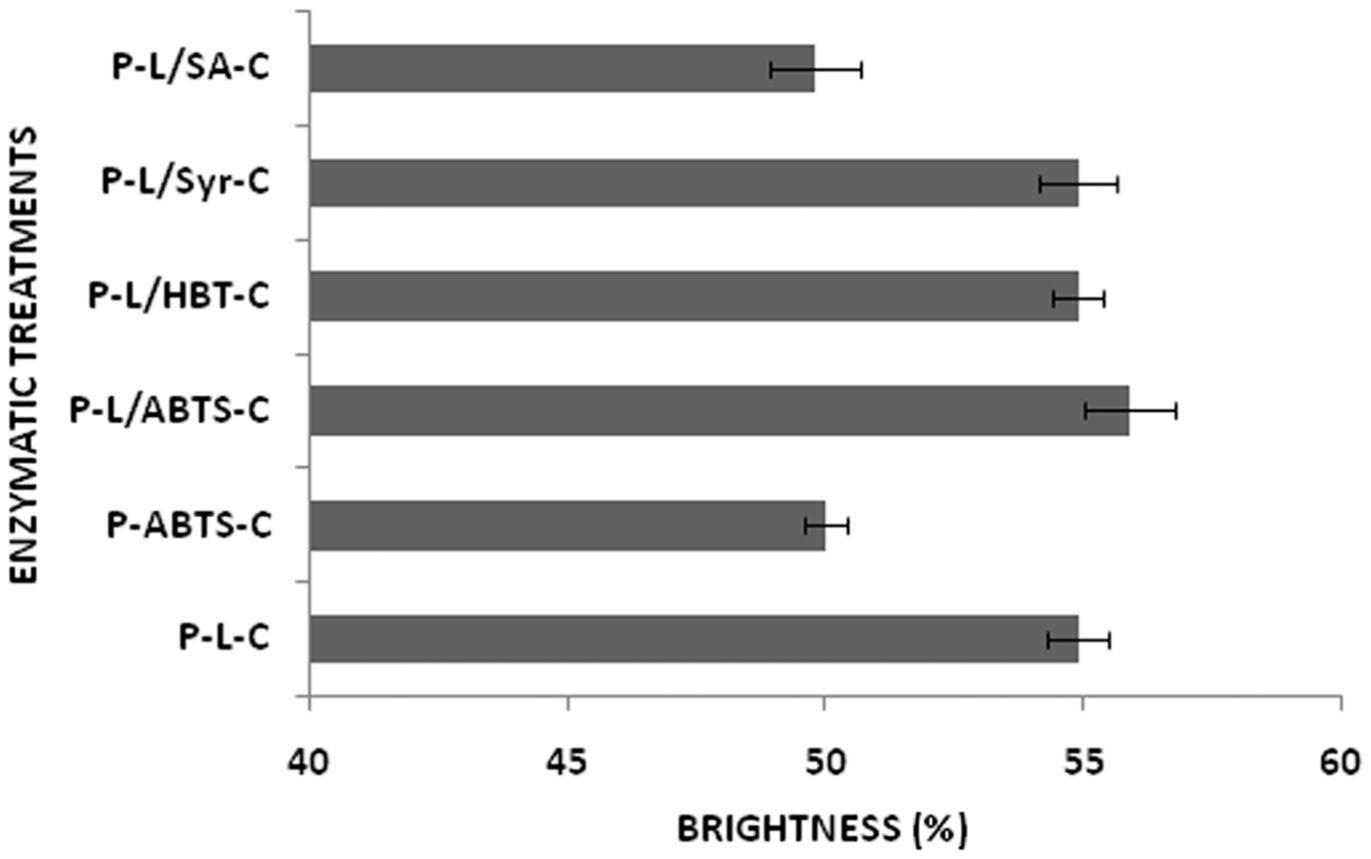
Effect of mediators (ABTS - 2,2′-azino-bis-3-ethylbenzothiazoline-6-sulphonic acid, HBT - 1-hydroxybenzotriazole, Syr - syringaldehyde) and inhibitor (SA – sodium azide) on the deinking of ONP pulp with laccase enzyme.

Different laccase mediators were tried to further enhance deinking (Figure 1). Except for ABTS, no other mediator showed any deinking effect either alone or in combination with laccase. However, ABTS supplementation showed only an additive effect when used with laccase rather than showing a mediating effect. Therefore, the bacterial laccase used in this study is being reported to require neither ABTS nor any other mediator for deinking, contrary to the previous reports [5], [11] where ABTS was found essential as a mediator for effective deinking of ONP pulp with laccase. ABTS acts as an electron shuttle between pulp fiber and the laccase molecule [24] and thereby causes deinking by oxidative action. A laccase requires a mediator only when its redox potential is less than that of the substrate to be oxidized. If the redox potential of laccase is already higher than the substrate (ink), then the need for mediator is eliminated.

Chemical deinking causes removal (dislodging) of ink particles from pulp fiber only whereas laccase enzyme may have caused either decolourization only or both decolourization and dislodging of ink particles. This was further supported by the fact that on comparing the absorbance scans of effluents (λ 200–800 nm) to detect the release of colored compounds, chemically treated pulp effluent showed high absorbance whereas laccase treated and untreated (control) pulp effluents were colourless (Figure S3).

This is the first report on deinking of ONP pulp with a bacterial laccase without the need of a mediator, thus making the process cost effective and eliminating mediator-linked enzyme toxicity problems. Furthermore, no deinking effect was observed with laccase in the presence of laccase inhibitor sodium azide which confirmed the role of laccase in deinking (Figure 1).

### Sequential Deinking of ONP Pulp with Xylanase and Laccase (P-X-L-C)

**Figure 2:**
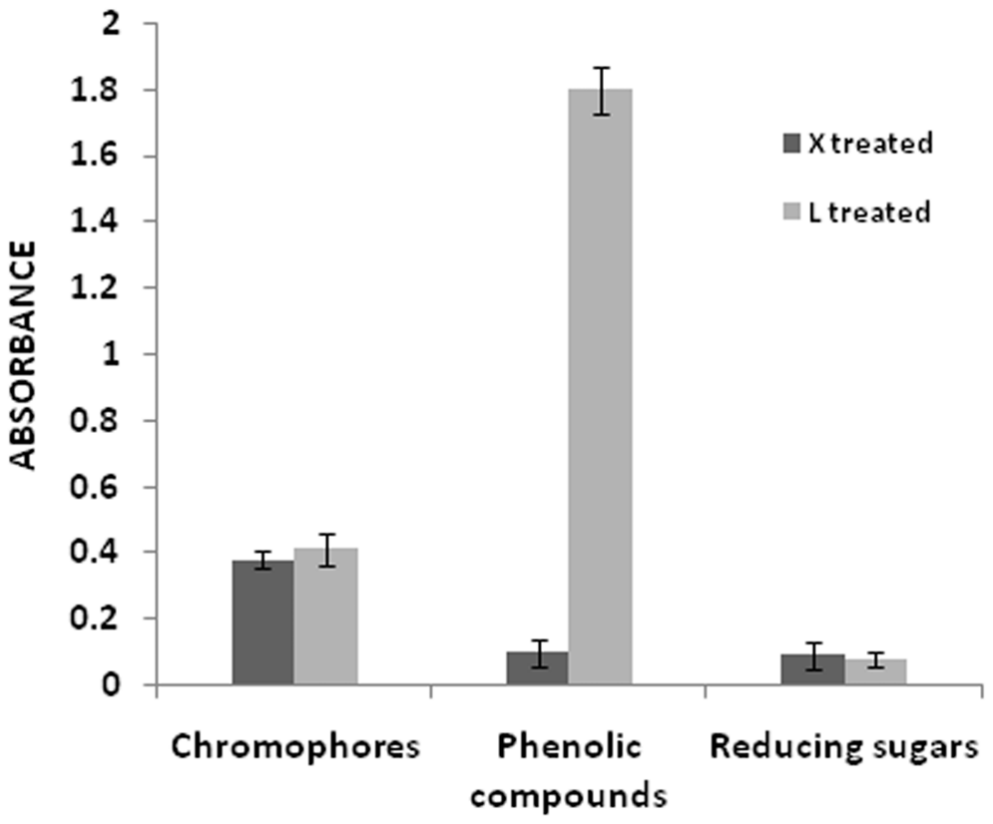
Analysis of phenolic compounds, hydrophobic compounds and reducing sugars in effluent released after xylanase (X) and laccase (L) treatment.

The present work demonstrated that an improved level of deinking was achieved by using xylanase and laccase sequentially as compared to xylanase or laccase alone. Pretreatment of ONP pulp with xylanase, followed by laccase, obtained a brightness of 59.6±0.8% ISO which was 8.5, 11.4 and 21.6% higher than that of the P-L-C, P-X-C and untreated pulp (Table 1). ERIC for P-X-L-C deinked pulp was 183.07±24 ppm which was 9.4, 35.2 and 65.8% lower than P-L-C, P-X-C and untreated pulp. The substantial reduction in residual ink concentration clearly showed the effective role of laccase followed by xylanase in deinking of ONP pulp. Xylanase acts on waste pulp, thereby removing xylan and opening the fibers for the subsequent chemical or enzyme attack. Laccase acts on the exposed lignin content of the pulp thereby facilitating the removal of lignin monomers and contaminants attached to lignin besides removing/dislodging ink particles. This synergistic action of xylanase and laccase thereby enhanced the brightness. Bacterial laccases have been previously reported to show potential in the biobleaching of pulp [25], [7], but not for their use in deinking of pulp. The correlation between release of chromophores (λ 237 nm), hydrophobic compounds (λ 465 nm), and reduction in kappa number coupled to the release of reducing sugars suggested the dissociation of lignin and sugars from pulp fibers (Figure 2). This work reports for the first time the use of a bacterial laccase alone and in combination with bacterial xylanase for deinking of pulp. The combined enzymatic treatment caused a substantial (50%) reduction in chemical consumption for deinking of ONP pulp (Table 2).

**Table 1:** Physical and Chemical characterization of enzymatically and physically deinked pulp.

Parameters	PC	PXC	PLC	PXLC	PSXLC	PMWXLC	PSMWXLC
**Kappa number**	10±0.9	9±0.78	8.8±0.74	8.1±0.83	7.8±0.75	7.7±0.75	7.4±0.66
**Brightness (% ISO)**	49.0±0.5	53.5±0.4	54.9±0.5	59.6±0.8	61.45±0.4	61.73±0.4	63.12±0.5
**Whiteness**	1.31±0.6	9.75±0.8	10.3±0.8	22.7±0.5	23.85±0.6	24.09±0.9	25.47±0.8
**ERIC (ppm)**	535.41±16	278.55±23	202.24±33	183.07±24	145.20±30	148.51±16	139.46±22
**Breaking Length (m)**	1285±29	1182±25	1378±22	1498±25	1328±32	1331±30	1345±33
**Burst Factor (kPa m^2^/g)**	6.12±0.8	6.34±0.56	6.27±0.62	6.38±0.58	6.24±0.9	6.26±0.7	6.3±0.5
**Tear Factor (mN m^2^/g)**	37.2±0.20	35.5±0.23	38.5±0.20	39.8±0.26	34.5±0.25	35.1±0.20	37.8±0.20
**Viscosity (cps)**	7.5±0.8	7.8±0.9	8.0±0.6	8.5±0.9	7.9±0.7	8.0±0.8	8.65±0.9

**Table 2:** Deinking of waste paper pulps by P-X-L-C treatment and reduction in chemical load.

Waste paper pulp		Brightness (%ISO)						
	P-C	P-X-L-C						
Chemicals used	100%	100%	90%	80%	70%	60%	50%	40%
**ONP print**	49±0.5	59.6±0.8	58.9±0.9	56.5±0.5	54.7±0.8	52.5±0.73	49.2±0.65	48.3±0.75
**Laser print**	81.5±0.4	84.1±0.5	82.8±0.8	82.3±0.3	81.5±0.7	79.2±0.6	–	–
**Magazines print**	51±0.5	55.24±0.7	52.5±0.9	52.2±0.5	50.9±0.5	48.9±0.4	–	–
**Xerox print**	85.6±0.4	87.3±0.5	86.8±0.4	86.1±0.8	85.7±0.7	84.1±0.4	–	–
**Inkjet print**	82±0.9	85.84±0.5	83.8±0.6	82.2±0.5	80.8±0.4	–	–	–

### X-ray Diffraction Analysis

**Figure 3:**
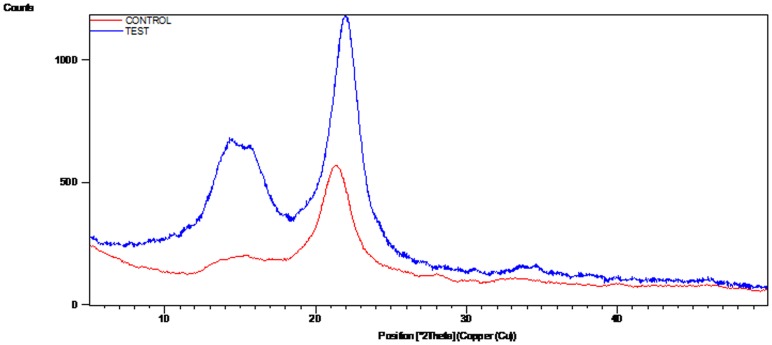
X-ray diffraction analysis of control (P-U-C) and test (P-X-L-C treated) ONP pulp.

The X-ray diffraction analysis of xylanase–laccase (P-X-L-C) treated ONP pulp is shown in Figure 3. The peak intensity from (002) lattice plane (2θ = 22.6°) represents the crystalline cellulose, while peak intensity of amorphous phase occur at 2θ = 19°. The I_002_ intensities (in cm) at 2θ = 22.6° were 2.4 and 8.4, I_am_ intensities (in cm) at 2θ = 19° were 1.7 and 2.9, half height width (in cm) of the peak angle of the (002) reflection at 2θ = 22.6° was 1.9 and 1.1 for the P-C and P-X-L-C pulps respectively. XRD analysis revealed that P-X-L-C treatment increased the crystallinity index of the ONP pulp from 29.16% (untreated) to 65.47% while the cellulose crystallinity index increased by 10.3% (Table 3) which means a decrease in amorphous cellulose and increase in crystalline cellulose of the pulp [26]. The increase in crystallinity might be due to removal of hemicelluloses and lignin and components adhered to lignin as a result of P-X-L-C treatment thereby increasing the cellulose content of the pulp [27]. The apparent cellulose crystal size of P-X-L-C treated pulp was altered by enzyme treatment showing 73% increase.

**Table 3:** The crystallinity index and apparent crystal size (ACS) of untreated (P-C) and treated (P-X-L-C) ONP pulp cellulose.

Parameters	P-C	P-X-L-C
Empirical crystalline index	29.16	65.47
Apparent crystal size (nm)	0.78	1.35
Cellulose crystallinity index (CI)	1.16	1.28

### FTIR Spectral Analysis

**Figure 4:**
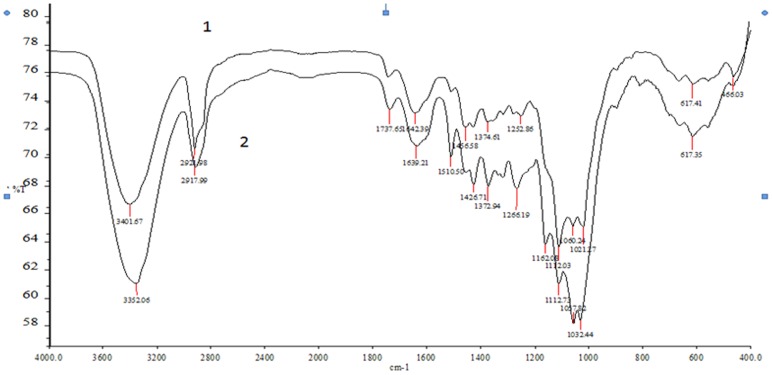
FTIR spectra of (1) P-C (2) P-X-L-C treated ONP pulp.

FTIR spectra for xylanase–laccase (P-X-L-C) treated pulp samples showed several characteristic and prominent changes (Figure 4). The P-X-L-C treated pulp showed absorption structures similar to P-C but with different intensities (Table 4). The bands around 3401–3352 cm^−1^ depict –OH stretching of hydrogen-bonding. The increase in their relative intensity after enzymatic treatments is attributed to the increase in cellulosic content of the pulp. The decrease in relative intensity of bands at 2921–2917 cm^−1^ and 617 cm^−1^, assigned to CH asymmetrical stretching vibration in CH_3_, CH_2_, CH, in P-X-L-C treated pulp indicated the degradation of aliphatic side chains. Relative intensity around 1642–1639 cm^−1^ increased in P-X-L-C treated pulp which is attributed to release of free carbonyl groups (C = O) due to action of enzyme on lignin’s aromatic ring [23]. In P-C pulp, these carbonyl groups remain associated with aromatic rings resulting in less absorption. Carboxylic acids appear to be intermediate degradation products in the complete mineralization of lignins to carbon dioxide and water [28]. The relative intensity of P-X-L-C pulp decreased at 1456–1426 cm^−1^, a band assigned to aromatic skeletal vibrations combined with –CH_3_ in-plane deformations, showing that some methoxyl groups were removed during the enzymatic treatment. The band at 1374–1372 cm^−1^ was assigned to aliphatic C–H stretching in CH_3_ (not in –OCH_3_) and phen–OH. The decrease of its relative intensity illustrated that either the side chains or phen–OH of lignin decreased after the enzymatic treatment. The decrease in relative intensity at 1266–1252 cm^−1^ indicates degradation of guaiacyl groups. A new band that appeared at 1737 cm^−1^ in P-X-L-C treated pulp was assigned to C = O stretching vibration in β-C = O, COOH, ester indicating that residual lignin after an P-X-L-C treatment was enriched in these types of functional groups. New band at 1162 cm^−1^ in P-X-L-C treated pulp indicated degradation of syringyl groups. The changes in pulp crystallinity derived from A_1430_/A_897_ ratio in accordance with the X-ray diffraction studies.

**Table 4:** Signals assignment of hand sheets in FTIR spectra and their relative intensity for untreated (P-C) and treated (P-X-L-C) pulp.

Bands cm^−1^	Assignment	Relative intensity	
		P-C	P-X-L-C
**3401–3352**	–OH stretching of hydrogen-bonding	1.28	1.36
**2921–2917**	CH asymmetrical stretching vibration in CH_3_, CH_2_, CH	1.06	0.95
**1737** *	C = O stretching vibration in β-C = O, COOH, ester	–	0.94
**1642–1639**	Aromatic skeletal vibrations plus C = O stretching	1.07	0.98
**1510**	Aromatic skeletal vibrations	1	1
**1456–1426**	Aromatic skeletal vibrations combined with –OCH_3_ in plane deformations	1.111	1.105
**1374–1372**	Aliphatic C–H stretching in CH_3_ and phen-OH	1.10	1.08
**1266–1252**	C-O stretching vibration in guaiacyl ring	1.13	1.11
**1162** *	C-H stretching vibration in syringyl ring	–	1.08
**1060–1057**	C-O bending vibration in primary alcohol, ether	1.38	1.47
**617** *	CH asymmetrical stretching vibration in CH_3_, CH_2_, CH	0.97	0.95

Relative intensity was calculated as the ratio of absorbance of the band to the absorbance of band at 1510 cm^−1^.

* Bands at 1737 and 1162 cm^−1^ appear only after enzymatic treatment.

### Scanning Electron Microscopy for Fiber Morphology

**Figure 5:**
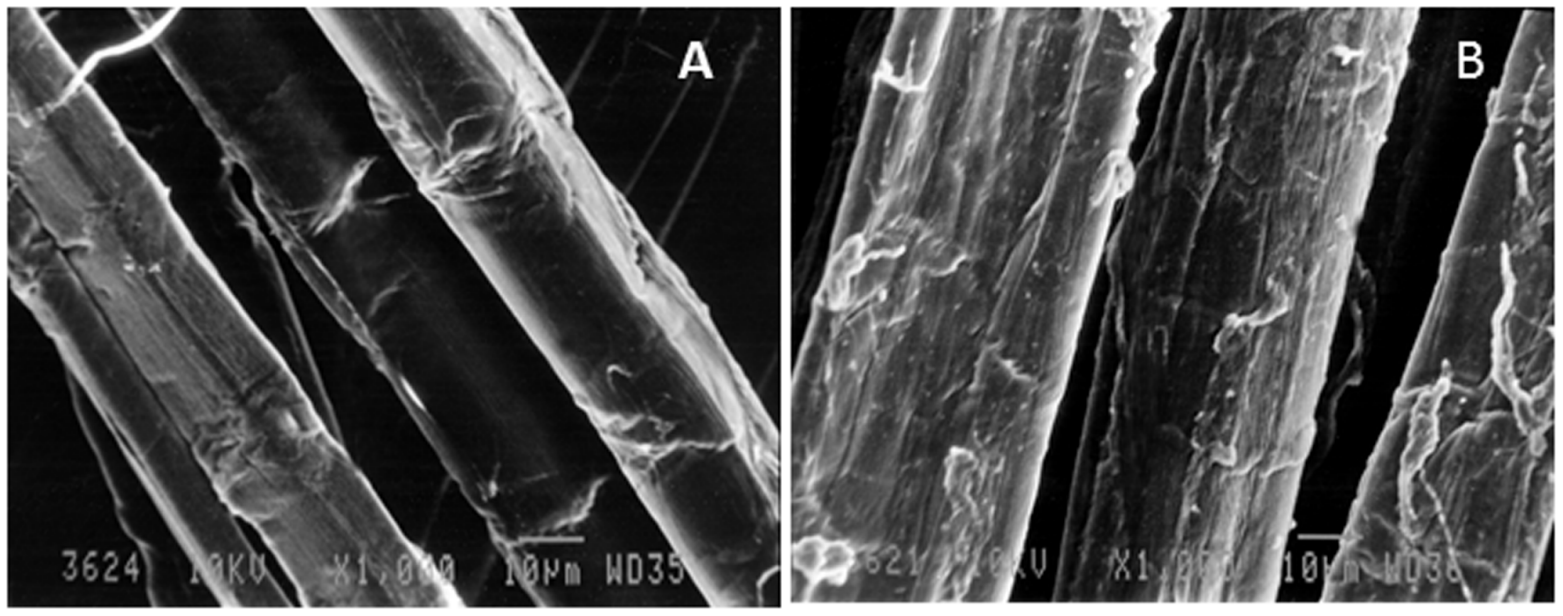
Scanning electron micrographs of (A) P-C and (B) P-X-L-C treated ONP pulp.

As can be observed from Figure 5, fiber surface of P-C pulp appears to be smooth. After P-X-L-C treatment the fiber surface became rough and fibrillation appeared on the surface indicating delignification on fiber surface which caused release of fibrils [29]. This is in accordance with the results of FTIR which shows that degradation of lignin occurred on the surface after enzymatic treatment. Other morphological changes like appearance of perforations and cracks probably occurred due to enzymatic hydrolysis by xylanase. These morphological changes increased the surface area of contact between chemicals and pulp fibers and resulted in reduced chemical consumption.

### Effect of Sonication (S) and Microwaving (MW) Pretreatments on Deinking of ONP

The parameters for the effect of sonication and microwave pretreatments alone and in combination with P-X-L-C pretreatment of ONP were optimized (Table 5). Maximum brightness of 52.1±0.6 and 52.8±0.41% ISO was obtained when sonication was done at 1% consistency, 230 W, 20 kHz for 15 min and when microwaving was done at 5% consistency, 850 W for 1 min.

**Table 5:** Optimization of parameters for sonication (S) and microwave (MW) treatment of ONP pulp.

SONICATION (S)			MICROWAVING (MW)		
	Brightness (%ISO)			Brightness (%ISO)	
Time (min)	P-S-C	P-S-X-L-C	Time (sec)	P-MW-C	P-MW-X-L-C
**0**	49±0.45	58.65±0.40	**0**	49±0.9	58.8±0.5
**5**	50±0.58	59.5±0.40	**30**	50.1±0.8	59.75±0.9
**10**	51.2±0.8	60.35±0.8	**60**	52.8±0.41	**61.73±0.33**
**15**	52.1±0.6	**61.45±0.75**	**90**	49.2±0.4	57.1±0.44
**20**	52±0.44	61.15±0.39	**120**	49.12±0.30	53.1±0.39

When used in combination with P-X-L-C pretreatment, further 3 (P-S-X-L-C) and 3.5% (P-MW-X-L-C) increase in brightness to 61.45±0.75 and 61.73±0.33% ISO was observed. Sonication causes swelling of fibers thus opening them which in turn increased the surface area of contact between fibers and enzymes or chemicals allowing them to reach protected inertial sites [30]. Sonication has been used previously for deinking of xerox print pulp showing a 10.39% increase in brightness [11]. Microwaving causes steam explosion thereby opening the fibers due to which hemicelluloses and lignin components get exposed to enzymes and chemicals [12]. It is also an efficient, fast, economic, easy and environmental friendly alternative to conventional heating of pulp for enzymatic or chemical bleaching. Microwaving has been explored previously in our lab for delignification of kraft pulp [13]. This study reports for the first time potential of microwave pretreatment for deinking purposes.

### Physical and Chemical Characterization of ONP Pulp (Table 1)

When all the three pretreatments (P-S-MW-X-L-C) were used together, the combination resulted in 62.13±0.5% ISO brightness (28.8% increase), 7.4±0.66 kappa number (22% decrease) and 139.04±22 ppm ERIC (73.9% reduction) as compared to brightness and ERIC of P-C pulps. Substantial reduction in ERIC to this level with the use of a new sequence combining enzymatic and physical methods for efficient deinking of ONP pulp has not been reported before. Though P-S-MW-X-L-C deinked pulp had lower strength properties than P-X-L-C deinked pulp, yet these were better than those of conventional chemically treated pulp. The breaking length of P-X-L-C deinked pulp was 26, 8.6 and 11% higher; burst factor was 0.6, 1.7 and 0.06% higher and tear factor was 12.1, 3.3 and 5.2% higher than P-X-C, P-L-C and P-S-MW-X-L-C deinked pulp. As none of the enzyme preparations had cellulase activity, a 13% increase in viscosity was observed in P-X-L-C treatment. P-L-C treatment caused 12% reduction in kappa number as compared to P-C, indicating the role of laccase in delignification.

### Deinking of other Wastepaper Pulps

Different wastepaper pulps were effectively deinked by using a combination of xylanase and laccase enzymes (Table 2). Maximum increase in brightness was obtained for ONP pulp (21.6%) followed by magazine pulp (8.3%), inkjet print pulp (4.1%), laser print pulp (3.1%) and xerox paper pulp (1.9%). This suggested that X-L system was able to remove both non-impact (newsprint) as well as impact (inkjet, laser and xerox) ink from waste papers. It was more effective in removing non-impact ink as this ink is not fused to the paper and gets easily removed in the deinking process. Impact ink is fused to the paper, hence being non-dispersible is difficult to remove [1]. A 30% reduction in chemical load with laser, magazines, xerox waste papers and 20% with inkjet waste paper was observed (Table 2).

### Conclusions

Laccase and xylanase effectively deinked wastepaper pulps without mediator supplementation for laccase activity. XL-pretreatment resulted in better optical and strength properties, highly reduced ERIC, higher cellulose crystallinity, more fibrillar surface and formation of lignin-degradation and conjugated carbonyl groups on the surface. A 50% reduction in chemical consumption for ONP pulp was also achieved. Sonication and microwaving contributed further to the improvement in the deinking of pulp. This work reports for the first time a new sequence combining biological and physical methods for effective deinking of old newsprint yielding better quality paper and substantially reduced requirement of chemicals.

## Supporting Information

Figure S1 **3D graph of combined effects of (A) Time and Enzyme dose (B) Time and pH (C) Enzyme dose and pH, on brightness when third factor was kept constant and its optimal level for xylanase treatment.**
(TIF)

Figure S2 **3D graph of combined effects of (A) Time and Enzyme dose (B) Time and pH (C) Enzyme dose and pH, on brightness when third factor was kept constant and its optimal level for laccase treatment.**
(TIF)

Figure S3 **Wavelength scan results for chemically treated (1), laccase treated (2) and untreated (3) ONP pulp.**
(TIF)

Table S1 **Experimental range and levels of independent test variables used in central composite rotary design for optimization of deinking of ONP pulp with xylanase or laccase.**
(DOC)

Table S2 **Central composite rotary design matrix with experimental values of brightness for optimization of pH, incubation time and enzyme dose for deinking of ONP pulp using xylanase and laccase enzymes.**
(DOC)

Table S3 **Response surface quadratic model analysis of variance (ANOVA) for deinking of ONP pulp using xylanase and laccase enzymes.**
(DOC)
