# Supplementary figures and images for: Combined Enzymatic and Physical Deinking Methodology for Efficient Eco-Friendly Recycling of Old Newsprint
Source: PLoS One. 2013 Aug 15;8(8):e72346. doi: 10.1371/journal.pone.0072346 (PMC3744503; doi:10.1371/journal.pone.0072346)

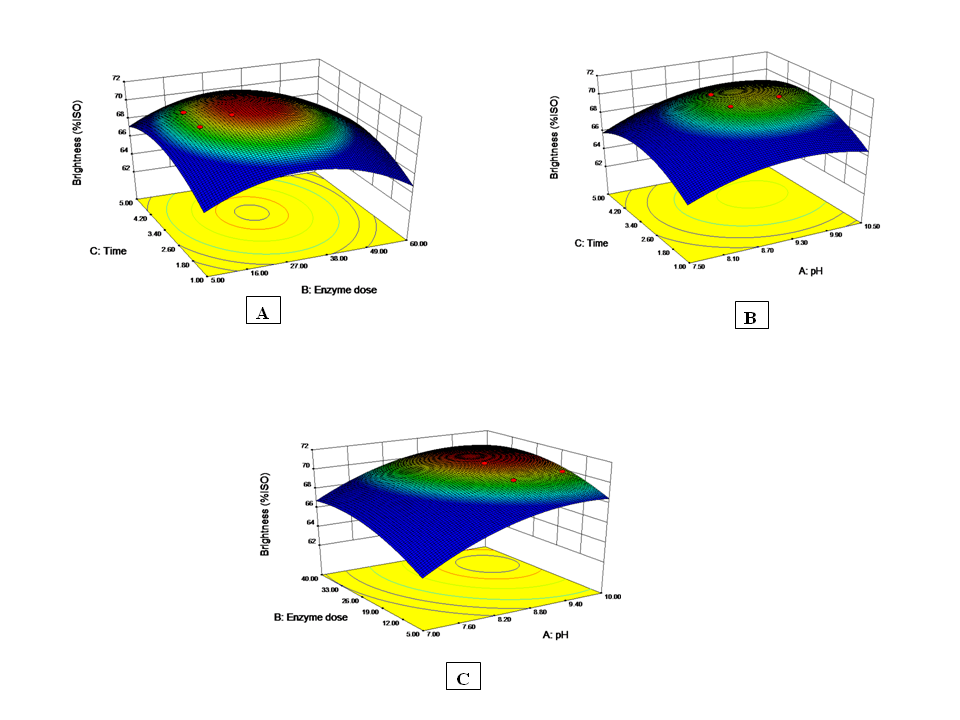

Supplement: Figure S1 — 3D graph of combined effects of (A) Time and Enzyme dose (B) Time and pH (C) Enzyme dose and pH, on brightness when third factor was kept constant and its optimal level for xylanase treatment. (TIF) [file pone.0072346.s001.tif]

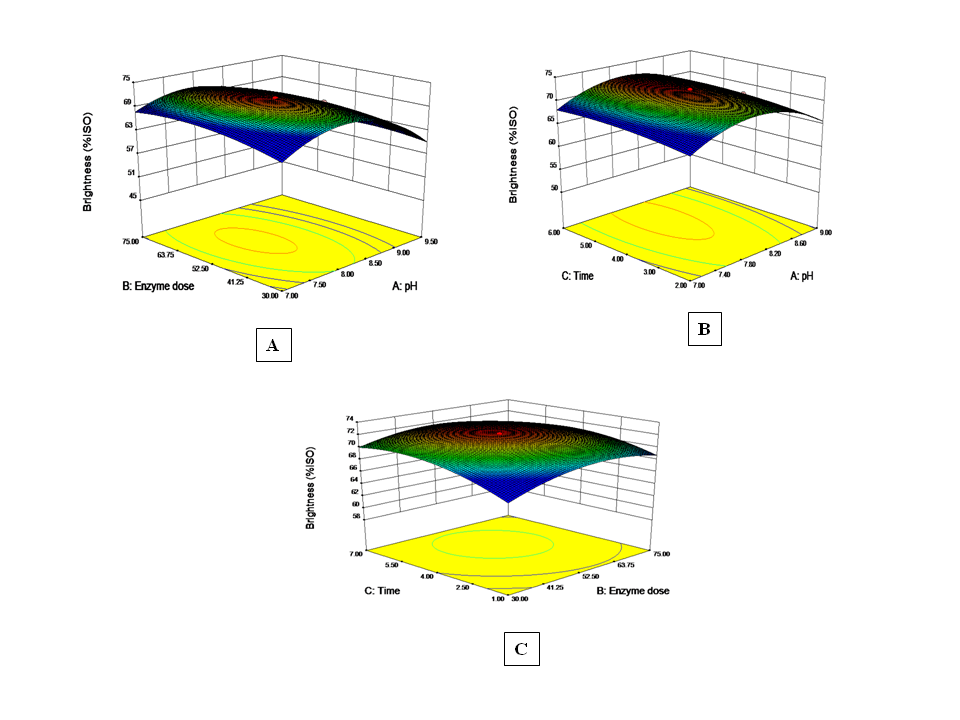

Supplement: Figure S2 — 3D graph of combined effects of (A) Time and Enzyme dose (B) Time and pH (C) Enzyme dose and pH, on brightness when third factor was kept constant and its optimal level for laccase treatment. (TIF) [file pone.0072346.s002.tif]

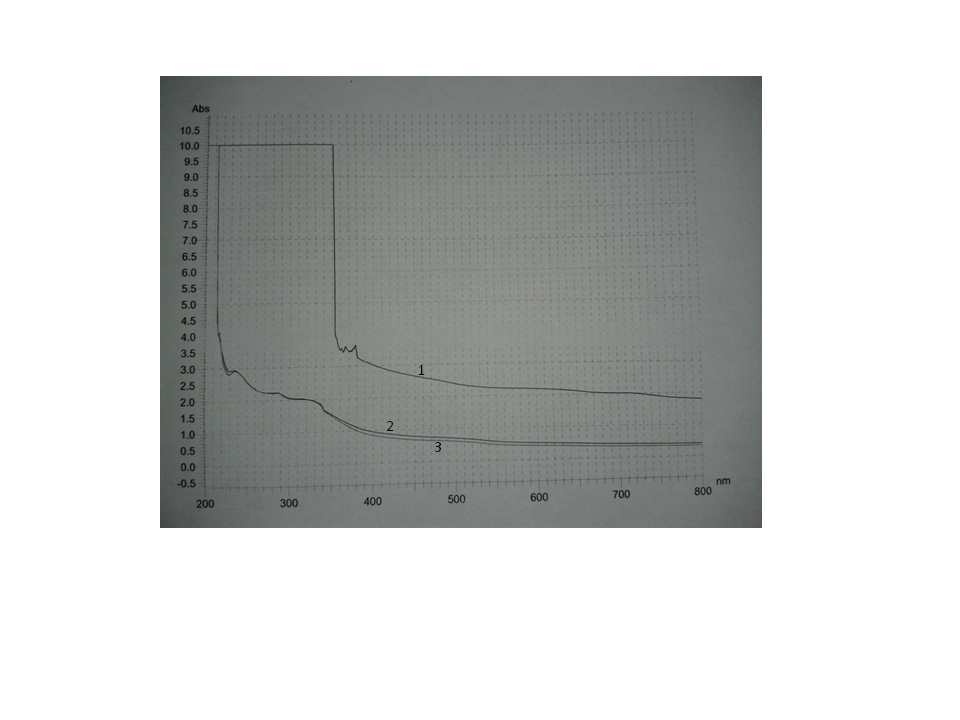

Supplement: Figure S3 — Wavelength scan results for chemically treated (1), laccase treated (2) and untreated (3) ONP pulp. (TIF) [file pone.0072346.s003.tif]
