# Supplementary material for: Combined Enzymatic and Physical Deinking Methodology for Efficient Eco-Friendly Recycling of Old Newsprint
Source: PLoS One. 2013 Aug 15;8(8):e72346. doi: 10.1371/journal.pone.0072346 (PMC3744503; doi:10.1371/journal.pone.0072346)
Supplement: Table S1 — Experimental range and levels of independent test variables used in central composite rotary design for optimization of deinking of ONP pulp with xylanase or laccase. (DOC) [file pone.0072346.s004.doc]

**Table S1** **Experimental range and levels of independent test variables used in central composite rotary design for optimization of deinking of ONP pulp with xylanase or laccase.**

| **Independent Variables** | **Level** | | | | |
| --- | --- | --- | --- | --- | --- |
|  | **-1.682** | **-1** | **0** | **1** | **1.682** |
| **pH** | 8.16 (7.16) | 8.5 (7.5) | 9 (8) | 9.5 (8.5) | 9.84 (8.84) |
| **Time (h)** | 2.16 (2.32) | 2.5 (3) | 3 (4) | 3.5 (5) | 3.84 (5.68) |
| **Enzyme dose (U/g odp)** | 6.59 (33.18) | 10 (40) | 15 (50) | 20 (60) | 23.41 (66.82) |

**Values for laccase treatment are given in parantheses**
