# Supplementary material for: Combined Enzymatic and Physical Deinking Methodology for Efficient Eco-Friendly Recycling of Old Newsprint
Source: PLoS One. 2013 Aug 15;8(8):e72346. doi: 10.1371/journal.pone.0072346 (PMC3744503; doi:10.1371/journal.pone.0072346)
Supplement: Table S2 — Central composite rotary design matrix with experimental values of brightness for optimization of pH, incubation time and enzyme dose for deinking of ONP pulp using xylanase and laccase enzymes. (DOC) [file pone.0072346.s005.doc]

**Table S2: Central composite rotary design matrix with experimental values of brightness for optimization of pH, incubation time and enzyme dose for deinking of ONP pulp using xylanase and laccase enzymes**

| **Run** | **pH** | **Xylanase/ Laccase dose** | **Time** | **Brightness (%ISO)** | |
| --- | --- | --- | --- | --- | --- |
|  |  |  |  | **Xylanase tretaed** | **Laccase treated** |
| **1** | -1 | -1 | -1 | 52.20±0.8 | 52.9±0.5 |
| **2** | 1 | -1 | -1 | 52.59±0.52 | 52.350.5 |
| **3** | -1 | 1 | -1 | 52.97±0.3 | 53.4±0.34 |
| **4** | 1 | 1 | -1 | 53.42±0.4 | 52.72±0.5 |
| **5** | -1 | -1 | 1 | 52.21±0.35 | 53.1±0.6 |
| **6** | 1 | -1 | 1 | 52.82±0.5 | 53.02±0.5 |
| **7** | -1 | 1 | 1 | 53.12±0.4 | 53.77±0.4 |
| **8** | 1 | 1 | 0 | 53.65±0.5 | 53.16±0.4 |
| **9** | -1.682 | 0 | 0 | 52.21±0.8 | 52.05±0.1 |
| **10** | 1.682 | 0 | 0 | 53.35±0.8 | 51.52±0.5 |
| **11** | 0 | -1.682 | 0 | 52.06±0.35 | 52.95±0.4 |
| **12** | 0 | 1.682 | 0 | 53.5±0.4 | 53.92±0.3 |
| **13** | 0 | 0 | -1.682 | 52.51±0.3 | 53.32±0.4 |
| **14** | 0 | 0 | 1.682 | 53.27±0.3 | 54.9±0.5 |
| **15** | 0 | 0 | 0 | 53.20±0.34 | 54.3±0.3 |
| **16** | 0 | 0 | 0 | 53.20±0.26 | 54.9±0.4 |
| **17** | 0 | 0 | 0 | 53.12±0.3 | 54.9±0.8 |
| **18** | 0 | 0 | 0 | 53.12±0.5 | 54.9±0.5 |
| **19** | 0 | 0 | 0 | 53.04±0.4 | 54.3±0.4 |
| **20** | 9 | 15 | 3 | 53.2±0.31 | 54.3±0.6 |
