# Supplementary material for: Combined Enzymatic and Physical Deinking Methodology for Efficient Eco-Friendly Recycling of Old Newsprint
Source: PLoS One. 2013 Aug 15;8(8):e72346. doi: 10.1371/journal.pone.0072346 (PMC3744503; doi:10.1371/journal.pone.0072346)
Supplement: Table S3 — Response surface quadratic model analysis of variance (ANOVA) for deinking of ONP pulp using xylanase and laccase enzymes. (DOC) [file pone.0072346.s006.doc]

**Table S3: Response surface quadratic model analysis of variance (ANOVA) for deinking of ONP pulp using xylanase and laccase enzymes.**

| **Source** | **Sum of squares** | **Df** | **Mean square** | **F value** | **p-value (Prob> F)** |  |
| --- | --- | --- | --- | --- | --- | --- |
| **Model** | 8.5 (24.91) | 9 (9) | 0.94 (2.77) | 46.86 (641.53) | <0.0001 (< 0.0001) | significant |
| **A- pH** | 2.07 (0.70) | 1 (1) | 2.07(0.70) | 102.93 (162.84) | < 0.0001 (<0.0001) |  |
| **B-Enzyme dose** | 5.25 (1.88) | 1 (1) | 5.25 (1.88) | 260.53 (435.71) | < 0.0001 (<0.0001) |  |
| **C-Time** | 0.53 (1.88) | 1 (1) | 0.53 (1.88) | 26.13 (435.71) | 0.0005 (<0.0001) |  |
| **AB** | 5.000E-003 (0.29) | 1 (1) | 5.000E-003 (0.29) | 0.25 (66.95) | 0.0292 (<0.0001) |  |
| **AC** | 5.000E-003 (8.000E-004) | 1 (1) | 5.000E-003 (8.000E-004) | 0.25 (0.19) | 0.0292 (0.6759) |  |
| **BC** | 0.000 (0.065) | 1 (1) | 0.000 (0.065) | 0.000 (15.02) | 1.0000 (0.0031) |  |
| **A^2^** | 0.42 (19.07) | 1 (1) | 0.42 (19.07) | 20.59 (4420.78) | 0.0011 (< 0.0001) |  |
| **B^2^** | 0.14 (2.00) | 1 (1) | 0.14 (2.00) | 7.01 (463.57) | 0.0244 (< 0.0001) |  |
| **C^2^** | 0.20 (0.55) | 1 (1) | 0.20 (0.55) | 9.73 (127.98) | 0.0109 (< 0.0001) |  |
| **Residual** | 0.20 (0.043) | 10 (10) | 0.020 (4.314E-003) |  |  |  |
| **Lack of fit** | 0.17 (0.028) | 5 (5) | 0.034 (5.627E-003) | 5.05 (1.88) | 0.0501 (0.2533) | not significant |
| **Pure error** | 0.033 (0.015) | 5 (5) | 6.667E-003 (3.000E-003) |  |  |  |
| **Cor total** | 8.70 (24.95) | 19 (19) |  |  |  |  |

***Values for laccase treatment are given in parantheses**
